# Medial calcaneal neuropathic pain clinically diagnosed in a child initially considered to have Sever’s disease: a case report

**DOI:** 10.1186/s40981-026-00871-7

**Published:** 2026-08-08

**Authors:** Katsuyuki Moriwaki, Rumiko Hachisuka, Sachiko Otsuki, Yasuo Tsutsumi

**Affiliations:** 1 Department of Anesthesiology, Hiroshima University Hospital, 1-2-3 Kasumi, Minami- ku, Hiroshima, 734-8551 Japan; 2 Department of Anesthesiology, Hiroshima Hiramatsu Hospital, Hiroshima, Japan

**Keywords:** Heel pain, Medial calcaneal neuropathy, Neuropathic pain, Sever’s disease, Allodynia, Pediatric chronic pain

## Abstract

**Background:**

Sever’s disease is a common cause of pediatric heel pain and usually improves with conservative treatment. However, persistent pain with allodynia and difficulty in heel contact may indicate a neuropathic mechanism.

**Case presentation:**

A 9-year-old boy developed persistent right heel pain after treatment for a suspected left calcaneal avulsion fracture. Despite a diagnosis of Sever’s disease and conservative therapy, pain, allodynia, and difficulty in heel contact persisted. The pain was localized to the medial and plantar heel, with a Tinel-like sign along the presumed course of the medial calcaneal nerve. Topical lidocaine–prilocaine cream and ultrasound-guided perineural injection of a local anesthetic temporarily relieved the allodynia and enabled heel contact. Ultimately, the symptoms resolved with multimodal treatment.

**Conclusions:**

Medial calcaneal neuropathic pain should be considered in children with persistent heel pain accompanied by sensory abnormalities, localized pain consistent with nerve distribution, and a Tinel-like sign.

## Background

Heel pain is a relatively common symptom in children, and Sever’s disease (calcaneal apophysitis) is one of the most common causes [1]. Sever’s disease is an overuse injury in which repetitive traction forces of the Achilles tendon and mechanical loading during growth result in microtrauma to the calcaneal apophysis. It may occur unilaterally or bilaterally and usually responds to conservative treatments, including activity modification, stretching, orthotic therapy, and rehabilitation [2, 3].

However, pediatric heel pain is not always attributable to Sever’s disease. Differential diagnoses include tendon disorders, stress fractures, infections, tumors, and nerve entrapment [4]. In particular, when heel pain is prolonged and accompanied by allodynia or sensory disturbance, a neuropathic cause, including medial calcaneal neuropathy, should be considered [4, 5].

Medial calcaneal neuropathy is a rare but important cause of chronic heel pain in adults [6, 7]. To our knowledge, no pediatric cases of medial calcaneal neuropathy have been reported. We report a pediatric case initially diagnosed as Sever’s disease but ultimately attributed to medial calcaneal neuropathic pain. This case highlights the importance of considering neuropathic pain in pediatric patients with persistent heel pain.

## Case presentation

A 10-year-10-month-old boy (height, 150.6 cm; weight, 50 kg) with no significant medical history fell while playing at the age of nine years and seven months. He underwent cast immobilization for approximately one month for a suspected avulsion fracture of his left calcaneus. Although the pain in the left heel improved, the pain in the contralateral right heel gradually worsened.

He was diagnosed with Sever’s disease at a local orthopedic clinic and received medication, a heel cup, stretching exercises, and muscle-strengthening exercises. However, the right heel pain persisted, and severe pain on heel contact led him to walk with an elevated heel.

Because the right heel pain persisted, he was referred to our orthopedic department and subsequently to our pain clinic five months before the current presentation for suspected complex regional pain syndrome (CRPS). Although allodynia was present, no vasomotor, sudomotor/edema, or motor/trophic signs or symptoms were observed; therefore, the patient did not fulfill the Budapest diagnostic criteria, and CRPS was considered unlikely. Therefore, treatment for Sever’s disease was continued.

Subsequently, the right heel pain worsened, interfered with school attendance, and was accompanied by severe abdominal pain. Therefore, the patient was referred to our department by the pediatric department of another general hospital.

**Fig. 1:**
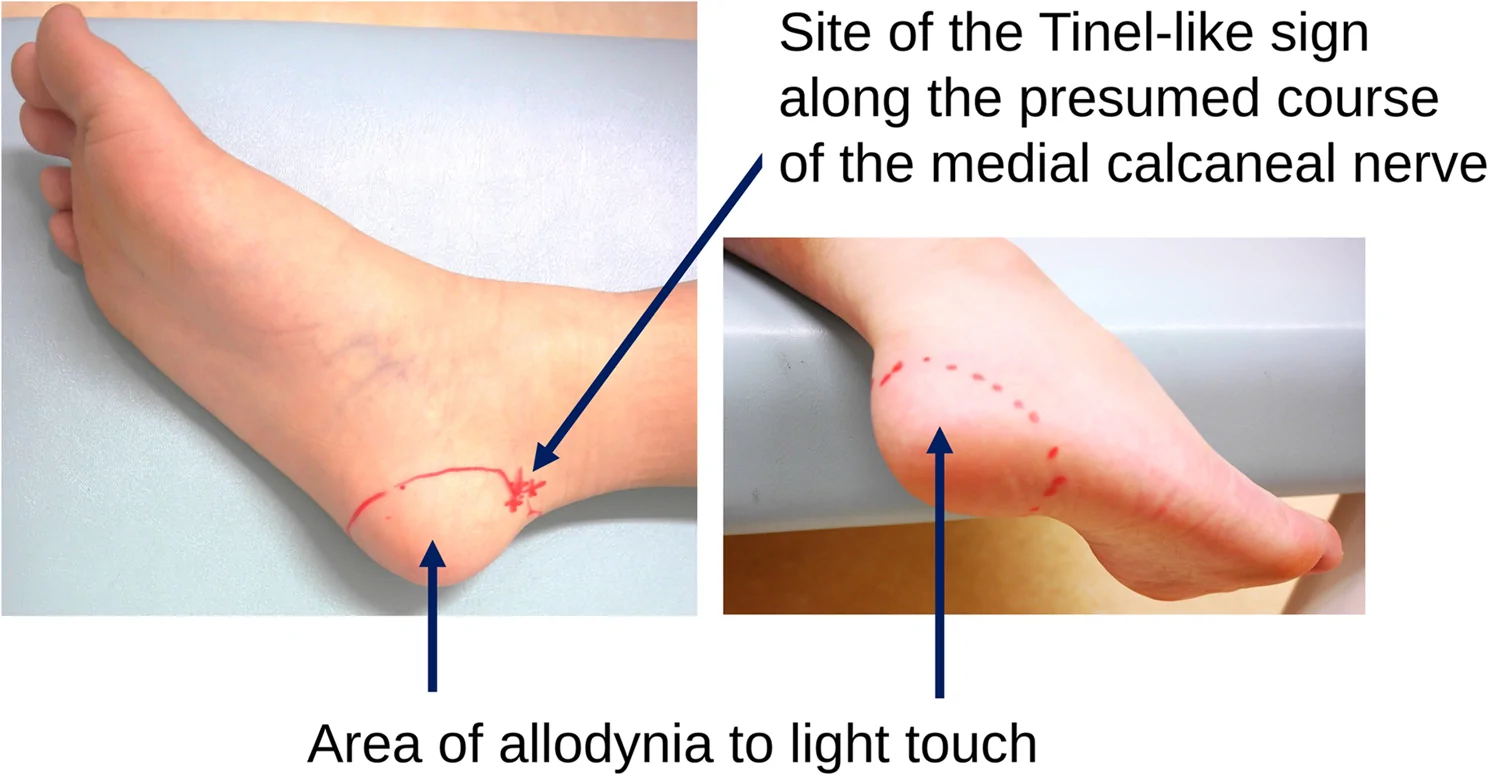
Localization of heel pain and Tinel-like sign along the presumed course of the medial calcaneal nerve. Marked allodynia elicited by gentle stroking with a cotton swab is observed in the medial and plantar aspects of the right heel, corresponding to the sensory distribution of the medial calcaneal nerve. The stimulation site is indicated by the red cross posteroinferior to the medial malleolus. Gentle repetitive tapping with a fingertip at this site reproducibly elicited radiating pain into the allodynic area of the heel. Arrows indicate the areas of allodynia and the site at which the Tinel-like sign is elicited

**Fig. 2:**
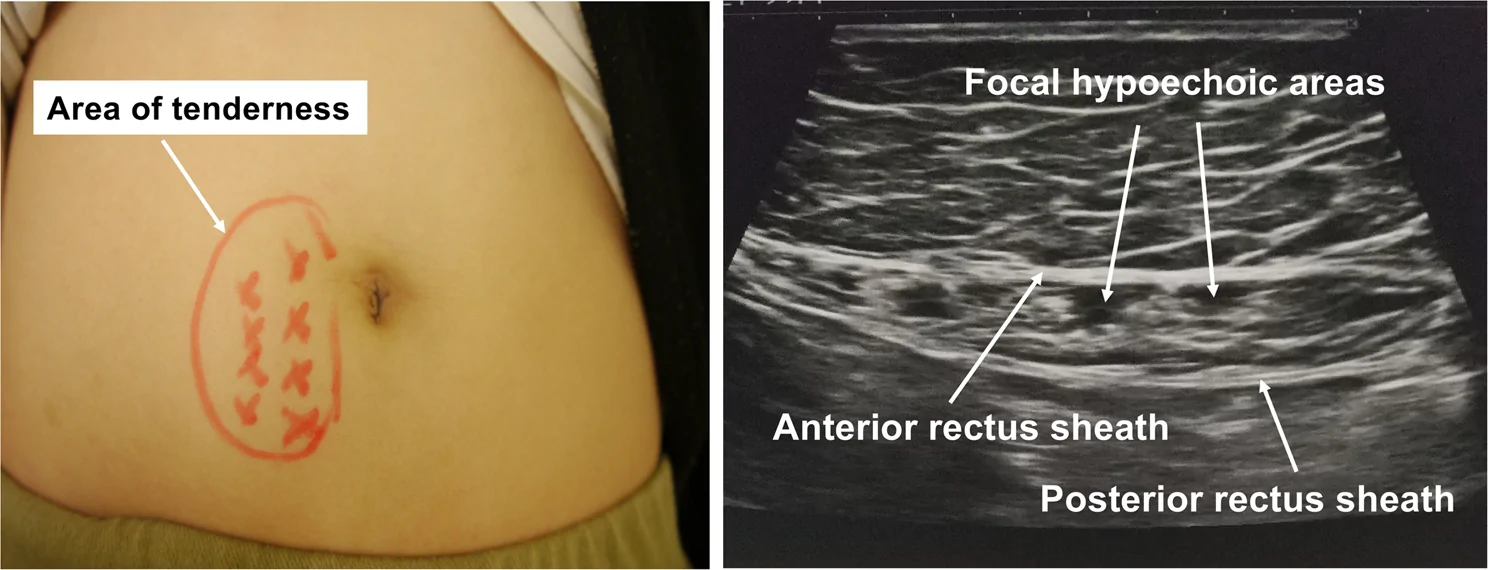
Location of abdominal tenderness and ultrasonographic findings of the right rectus abdominis muscle. The area of tenderness was localized within the right rectus abdominis muscle, without tenderness lateral to the muscle. Ultrasonography demonstrated focal hypoechoic areas within the muscle at the tender site

At re-referral, pain prevented right heel contact (Fig. 1). Abdominal pain worsened after meals and lasted for 15–30 min. Upper gastrointestinal endoscopy performed at a previous hospital revealed no significant abnormalities. Physical examination revealed localized tenderness at the umbilical level of the right rectus abdominis muscle, without tenderness lateral to the muscle or sensory disturbance. Ultrasonography showed focal hypoechoic areas within the muscle at the tender site (Fig. 2). Ultrasound-guided trigger point injection of 7 mL of 1% mepivacaine at this site resulted in complete but temporary resolution of the abdominal pain. These findings suggested the possibility of rectus abdominis myofascial pain.

Marked allodynia was elicited by gentle stroking of the right heel with a cotton swab [8] (Fig. 1). Because a neuropathic component was suspected, amitriptyline was initiated, followed by the addition of pregabalin. Neurological evaluation revealed no evidence of central nervous system pathology. A Tinel-like sign was most prominent along the presumed course of the medial calcaneal nerve near the Achilles tendon (Fig. 1). Further orthopedic evaluation, including magnetic resonance imaging, revealed no structural abnormalities requiring surgical decompression.

**Fig. 3:**
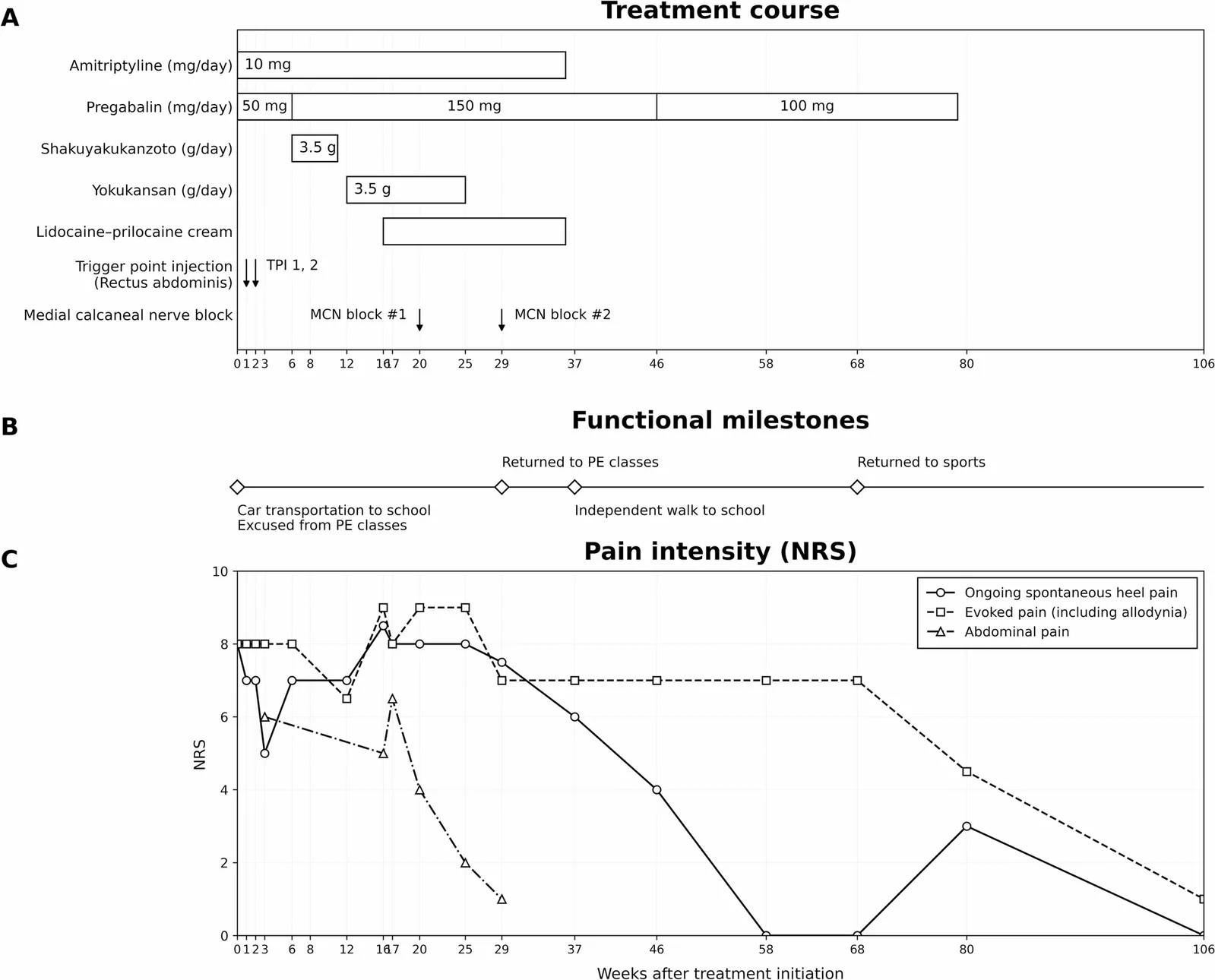
Treatment course, functional recovery, and pain intensity over time. Panel **A**: Timeline of pharmacological treatment and interventional procedures. Amitriptyline, pregabalin, shakuyakukanzoto, yokukansan, and lidocaine–prilocaine cream were administered during the periods indicated. Trigger point injections to the right rectus abdominis muscle and two medial calcaneal nerve blocks, performed as ultrasound-guided nerve-targeted injections adjacent to the presumed course of the medial calcaneal nerve, were performed at the time points shown. Panel **B**: Functional milestones illustrating the patient’s progressive recovery. Panel **C**: Changes in pain intensity for ongoing spontaneous heel pain, evoked heel pain including allodynia, and abdominal pain over time. Abbreviations: MCN, medial calcaneal nerve; NRS, numerical rating scale; PE, physical education; TPI, trigger point injection

The abdominal pain gradually improved after two trigger point injections into the right rectus abdominis and oral treatment with amitriptyline, pregabalin, shakuyakukanzoto, and yokukansan (Fig. 3). The abdominal pain score decreased over the first 29 weeks after treatment initiation, during which tenderness with a palpable taut band of the right rectus abdominis muscle almost disappeared. By 37 weeks after treatment initiation, the abdominal pain had completely resolved (Fig. 3).

Four months after treatment initiation, because the heel pain persisted, lidocaine–prilocaine cream (EMLA^®^ cream; AstraZeneca, UK), a topical local anesthetic used for cutaneous analgesia, was applied, which temporarily alleviated the allodynia and pain on heel contact. Under ultrasound guidance, 2 mL of 1% mepivacaine and 10 mg of triamcinolone were injected adjacent to the presumed course of the right medial calcaneal nerve at the site of the most prominent Tinel-like sign near the Achilles tendon. This procedure was performed on two occasions following the application of lidocaine–prilocaine cream (Fig. 3). Following the injection, the allodynia of the right heel almost completely disappeared, and the patient was temporarily able to stand with heel contact with the ground. Based on these findings, medial calcaneal neuropathic pain was considered the primary cause of the persistent right heel pain.

Although the distribution of allodynia remained almost unchanged until week 68, the intensity of evoked pain, including allodynia, gradually decreased (Fig. 3). As the patient’s activity level improved, pregabalin was gradually tapered and discontinued 80 weeks after the initiation of treatment (Fig. 3).

At that time, heel contact pain improved to a tolerable level, allowing him to run and participate in normal school activities (Fig. 3). At the final visit, 106 weeks (approximately 2 years) after treatment initiation, at age 12 years 11 months (170 cm), he stated that he was “mostly fine.” According to his mother, he had practiced judo barefoot for the previous two months. The numerical rating scale (NRS) scores for spontaneous and evoked heel pain improved to 0 and 1, respectively (Fig. 3), and the allodynia completely resolved.

Written informed consent was obtained from the patient and his guardian for the publication of this case report and the accompanying images. The off-label use of pregabalin and lidocaine–prilocaine cream was classified as Risk B under the institutional Handling Standards for Unapproved and Novel Drugs and Devices. This classification requires review and approval by the hospital pharmacy department. Approval was obtained for each drug in accordance with this institutional process. Informed consent for the off-label use of each drug was obtained from the patient and his legal guardian, separately from the consent for publication of this case report. No clinically significant adverse effects attributable to pregabalin or the other medications were observed during the treatment course.

## Discussion

Although Sever’s disease is the most common cause of pediatric heel pain and typically improves with conservative treatment [1–3], atypical features such as persistent pain, allodynia, and difficulty in heel contact should prompt reconsideration of the diagnosis. This case suggests that medial calcaneal neuropathic pain may be an under-recognized cause of persistent heel pain in children initially diagnosed with Sever’s disease. The clinical features distinguishing medial calcaneal neuropathic pain from typical Sever’s disease are summarized in Table 1.

**Table 1:** Comparison of the characteristic clinical features of medial calcaneal neuropathic pain and Sever’s disease with the findings in the present case

Clinical feature	Medial calcaneal neuropathic pain [5]	Sever’s disease (calcaneal apophysitis) [2]	Present case
Pain location	Medial heel and posteromedial plantar aspect of the heel, corresponding to the sensory distribution of the medial calcaneal nerve	Posterior heel, particularly around the calcaneal apophysis and Achilles tendon insertion	Consistent with medial calcaneal nerve distribution
Pain characteristics	Neuropathic pain, such as burning, electric shock-like pain, or numbness	Activity-related heel pain	Neuropathic features present
Relationship to activity	May be aggravated by weight-bearing and walking; pain may also occur at rest or during the night	Aggravated during or after physical activity, particularly running and jumping, and relieved by rest	Pain during walking and at rest
Sensory abnormalities	May be present, including allodynia, dysesthesia, or hypoesthesia	Typically absent	Allodynia present
Tenderness	Symptoms are reproduced or aggravated by pressure along the course of the medial calcaneal nerve	Localized tenderness over the posterior calcaneus, particularly at the Achilles tendon insertion; pain may be reproduced by the calcaneal squeeze test	Tenderness along the medial calcaneal nerve course
Tinel-like sign	May be elicited along the course of the medial calcaneal nerve, typically posteroinferior to the medial malleolus	Not a characteristic finding	Present

The characteristic clinical features of medial calcaneal neuropathic pain and Sever’s disease were summarized from Brown et al. [5] and Smith and Varacallo [2], respectively. The rightmost column shows the corresponding findings in the present case.

Several findings support a neuropathic mechanism. The pain was localized to the medial and plantar aspects of the heel and corresponded to the sensory distribution of the medial calcaneal nerve. Tactile allodynia and a Tinel-like sign were observed along the presumed nerve course. In addition, both topical anesthetic application and perineural injection resulted in clear, although temporary, relief of symptoms and restoration of heel contact. These findings are consistent with those of peripheral neuropathic pain.

The medial calcaneal nerve is a sensory branch of the posterior tibial nerve that innervates the medial and plantar aspects of the heel [5]. Its anatomical variability and course through mechanically vulnerable regions make it susceptible to compression, traction, and repetitive microtrauma [9, 10]. Even in adults, medial calcaneal neuropathy may be overlooked and misdiagnosed as plantar fasciitis or other common causes of heel pain [6, 7]. This case suggests that medial calcaneal neuropathy should be considered in pediatric patients, particularly when the clinical features are inconsistent with those of typical Sever’s disease (Table 1).

The characteristic forefoot-loading gait observed in this patient may have contributed to symptom persistence. As the medial calcaneal nerve can be affected by foot alignment abnormalities, compression, and repetitive microtrauma [5], pain-avoidance toe walking may have altered the mechanical environment around the heel and increased nerve irritation. Medial calcaneal neuropathy has also been reported in association with high-heeled shoe use [11].

Another notable feature was abdominal wall pain involving the right rectus abdominis region. Rectus abdominis myofascial pain was considered on the basis of the localized muscle tenderness, ultrasonographic findings, and temporary response to trigger point injection. However, anterior cutaneous nerve entrapment syndrome could not be completely excluded because Carnett’s sign and the pinch test were not assessed, and spread of the local anesthetic to a nearby anterior cutaneous nerve could not be ruled out.

A previous case report suggested an association between lower-limb structural imbalance and abdominal wall pain [12]. In the present case, prolonged toe walking and an asymmetric gait might have contributed to rectus abdominis overactivity. Additionally, food intake induces an abdominal accommodation response characterized by diaphragmatic relaxation and contraction of the upper anterior abdominal wall muscles, particularly the rectus abdominis and external oblique muscles [13]. Postprandial exacerbation of abdominal pain might also be partly explained by increased activation of these muscles; however, this mechanism remains speculative.

This study has some limitations. The diagnosis of medial calcaneal neuropathic pain was based on clinical findings and treatment response without electrophysiological confirmation or direct imaging evidence of nerve pathology. Although recent studies have demonstrated that high-resolution ultrasonography can visualize the medial calcaneal nerve and may assist in diagnosis and treatment [14], the nerve itself was not clearly visualized in this patient. Therefore, in this patient, medial calcaneal neuropathic pain was clinically diagnosed on the basis of the characteristic pain distribution, allodynia, a Tinel-like sign, and the response to local anesthetic administration. Furthermore, the possibility of coexisting Sever’s disease in the early phases cannot be excluded. Symptom improvement may also be influenced by multiple factors, including pharmacotherapy, local interventions, rehabilitation, and possible growth-related changes.

In conclusion, medial calcaneal neuropathic pain should be considered in the differential diagnosis of pediatric patients with persistent heel pain accompanied by allodynia and difficulty in heel contact.

## Data Availability

The datasets generated and/or analyzed during the current study are available from the corresponding author on reasonable request.

## Abbreviations

CRPS: Complex regional pain syndrome
EMLA: Eutectic mixture of local anesthetics (lidocaine–prilocaine cream)
MRI: Magnetic resonance imaging
NRS: Numerical rating scale
TPI: Trigger point injection
